# Viability of equine mesenchymal stem cells during transport and implantation

**DOI:** 10.1186/scrt483

**Published:** 2014-08-08

**Authors:** Elaine R Garvican, Sandra Cree, Lydia Bull, Roger KW Smith, Jayesh Dudhia

**Affiliations:** The Royal Veterinary College, Clinical Sciences and Services, Hawkshead Lane, North Mymms, Hatfield AL9 7TA UK

## Abstract

**Introduction:**

Autologous mesenchymal stem cell (MSC) injection into naturally-occurring equine tendon injuries has been shown to be safe and efficacious and protocols inform translation of the technique into humans. Efficient transfer of cells from the laboratory into tissue requires well-validated transport and implantation techniques.

**Methods:**

Cell viability in a range of media was determined over 72 hours and after injection through a 19G, 21G or 23G needle. Viability, proliferation and apoptosis were analysed using TrypanBlue, alamarBlue^®^ and AnnexinV assays.

**Results:**

Cell viability was similar in all re-suspension media following 24 hour storage, however cell death was most rapid in bone marrow aspirate, platelet-rich plasma and serum after longer storage. Cryogenic media exhibited greatest viability regardless of storage time. Cell proliferation after 24 and 72 hour storage was similar for all media, except after 24 hours in serum wherein proliferation was enhanced. MSC tri-lineage differentiation and viability did not significantly change when extruded through 19G, 21G or 23G needles, but 21G and 23G needles significantly increased apoptotic cells compared to 19G and non-injected controls. All gauges induced a decrease in metabolic activity immediately post-injection but cells recovered by 2 hours.

**Conclusions:**

These data indicate storage and injection influence viability and subsequent cell behaviour and provide recommendations for MSC therapy that implantation of cells should occur within 24 hours of recovery from culture, using larger needle bores.

## Introduction

The use of autologous mesenchymal stem cells (MSCs) derived from bone marrow to enhance tendon lesion repair is supported by a growing body of evidence from both experimental and clinical studies [1–5]. In the tendon, influx of a local subpopulation of precursor cells is believed to provide intrinsic post-injury repair [6]. However, these cells appear to be largely derived from peri-tendinous origins such as the paratenon [7] and result in fibrous repair with mechanical, structural and compositional differences from normal tendon. In an attempt to modify the repair towards regeneration, enhancing the small numbers of endogenous MSCs by implantation of large numbers of autologous, culture-expanded MSCs derived from donor tissue such as bone marrow, has been proposed. This hypothesis has been supported by positive results in experimental laboratory animal models of Achilles and patellar tendon injury [4, 5]. However, many laboratory animal models of induced injury have limited relevance to the human disease [8].

Horses, in contrast, suffer naturally-occurring flexor tendon injury with many similarities to human Achilles tendinopathy, making the horse a useful translational model for stem cell therapies. In recent years, a methodology for isolation, expansion and injection of autologous bone marrow-derived MSCs into lesions of the equine superficial digital flexor tendon has been widely accepted into clinical practice for the treatment of this disease [9]. Optimisation of cell-based therapies would ideally require accurate delivery to the target area without significant loss of cellular function or viability [10, 11], although a recent study established that only 24% of injected MSCs were retained at the site of injury after 24 hours [12]. Factors that may influence this poor cell retention include reduced cell viability following transport of the cells to the clinic or damage during the process of intralesional injection.

The current, commercial application of MSCs involves laboratory proliferation to achieve sufficient cells that then are either transported overnight, at 4 to 8°C, in autologous bone marrow aspirate (BMA) at a concentration of 5 × 10^6^ cells/ml [1] or are frozen [13]. The former technique was designed to achieve a fully autologous preparation that can be immediately injected, while the second requires thawing before implantation. These protocols, however, have not been tested in comparison with each other, or with other potential transport media. The first aim of this study was therefore to evaluate cell survival in different transport media.

Once the cells arrive at the clinic, they are injected under ultrasound guidance directly into the lesion within the recipient tendon. The disrupted central area of the tendon provides a cavity into which the cells are slowly injected with minimal injection pressure. Cells respond to external mechanical influences that affect their survival, growth and differentiation. Inappropriate stimulation of signalling pathways can lead to premature apoptosis, a complex cascade of events leading to the final demise of the cell [14]. The injection process could therefore potentially influence post-injection cell survival and metabolism. The most likely cause of cellular damage during injection is sheer stress, caused by the turbulent flow of fluid at the outer edges of the flow channel, where viscous fluid is in contact with the channel walls. Sheer stress is influenced by pressure, cross-sectional area and length of the channel [15], and by sudden changes in geometry, such as the rapid tapering of the vessel diameter from syringe to needle hub [10]. The surface marker phenotype and differentiation ability of rat and human MSCs were reportedly unaffected immediately following injection through catheters and needles at clinically relevant sizes and flow rates [10, 16], although a significant reduction in viability was observed for all catheters 24 hours after infusion. In contrast, another study demonstrated that longer and thinner cannulae were damaging to cells [17]. Other studies investigating cell printing methods to deposit cells in tissue scaffolds demonstrated significant loss of viability with increasing dispensing pressure and reducing nozzle size [18, 19] and suggested that a recovery period was necessary following injection. The technique developed for clinical use in the horse, which has direct relevance to human treatment, utilises a 2 ml syringe and hypodermic needle. A second aim of the present study was therefore to investigate the influence of needle size in this system on both cell viability and the potential delayed effects of injection.

This study demonstrates that transport media but not needle size influenced cell viability, but both had delayed effects on cell metabolism and apoptosis.

## Materials and methods

### Isolation of mesenchymal stem cells

Ethical approval for the collection of bone marrow aspirates and blood was received from the Ethics and Welfare Committee at the Royal Veterinary College (URN 2013 1230R 2005). No horses were euthanased for the sole purpose of obtaining tissues for this study. Bone marrow-derived MSCs (*n* = 3) were obtained and expanded as described previously [9] and P0 to P2 passage cells were stored in Bambanker™ cell freezing medium (Anachem, Luton, UK) in liquid nitrogen until use. For experiments, cells were seeded in D10 medium (Dulbecco’s modified Eagle’s medium, supplemented with foetal calf serum (10% v/v), 100 U/ml penicillin, and 100 U/ml streptomycin; all from Invitrogen, Paisley, UK) and were expanded to the required numbers. Cells were then detached from the culture flasks by trypsin–ethylenediamine tetraacetic acid (Sigma-Aldrich, Gillingham, UK) for storage media and needle gauge experiments.

### Effect of storage media

MSCs (2.5 × 10^6^ cells) were resuspended in the test media at a concentration of 5 × 10^6^ cells/ml and transferred to 1 ml cryotube vials (PAA, Yeovil, UK). Resuspension media were as follows: D10; allogenic BMA; hyaluronic acid (as sodium hyaluronate at a concentration of 10 mg/ml in isotonic sodium chloride–phosphate buffer, pH 7.0; Bayer PLC, Newbury, UK); allogenic plasma (heparinised) and allogenic serum obtained by routine venipuncture from a horse free of systemic disease; isotonic saline (Dechra Pharmaceuticals, Northwich, UK); allogenic platelet-rich plasma (prepared by routine venipuncture into 3.8% trisodium citrate (ratio 9:1), after which platelets were concentrated by centrifugation as previously described [20] and the platelet pellet was resuspended in D10 (2 ml for platelets pellets prepared from 20 ml plasma)); and cryogenic cell freezing medium (90% equine serum, 10% dimethyl sulphoxide).

Vials were stored at 4 to 8°C until analysis except for those containing cell-freezing medium, which were stored in dry ice (-78°C). Prior to assaying, these vials were warmed briefly in a 37°C water bath with care to avoid heating beyond melting point. Cells were thawed by slow addition (over 90 seconds) of an equal volume of lactated Ringer’s solution (Dechra Pharmaceuticals) to the vial. After storage for 12, 24, 48 and 72 hours, cells for all media were assayed for viability and cell proliferation as detailed below.

### Effect of needle gauge

MSCs were diluted to a final suspension density of 5 × 10^6^ cells/ml in D10. One millilitre of cell suspension from each cell line was set aside as a non-injected control group. One-millilitre aliquots of MSCs were slowly aspirated into a 2 ml syringe with the appropriate test needle attached (as is the practice for clinical MSC injection). Needle sizes of 21G (currently used for MSC injection), 23G (one gauge smaller) and 19G (one gauge larger) were used. All needles were 50 mm in length (Terumo Ltd, Bagshot, UK). The loaded 2 ml syringe was then fixed concentrically within a 30 ml syringe to enable mounting into a Flo-Gard GSP syringe pump (Baxter, Newbury, UK). The extrusion rate was set to 900 ml/hour and the volume (Volume to be Infused) set at 7.5 ml, which was appropriate to deliver a 1 ml volume from the 2 ml syringe over 30 seconds to mimic the slow, steady injection of MSCs applied in clinical practice. Cell viability, proliferation and apoptosis assays were performed immediately after extrusion of cells from the needles and on the non-injected control cells.

### Viability assay

The viability of cell suspensions from both the storage media and needle experiments were determined by mixing 100 μl cell suspension with 100 μl of 0.4% trypan blue solution (Sigma-Aldrich) for 2 minutes. Cells were counted using a haemocytometer microchamber under a light microscope [21].

### Cell proliferation assay

To assess the ability of the cells to proliferate following storage in the various storage media, a proliferation assay was performed using the alamarBlue^®^ assay (AbD Serotec, Kidlington, UK). Then 5 × 10^3^ cells were seeded in duplicate into each well of a microtitre plate in D10 and allowed to adhere for 24 hours. The media were replaced with 1 ml D10 containing 100 μl alamarBlue^®^ reagent and cells were incubated for a further 4 hours, shielded from light. Then 100 μl aliquots were transferred to a black fluoro-microtitre plate (SPL LifeSciences, Singapore, UK) and fluorescence was measured at 570 nm (excitation) and 585 nm (emission) (Infinite M200 PRO fluorometer; Tecan, UK). The media in the cells was replaced with fresh D10 and the assay was repeated at 24, 48 and 72 hours as above.

### Mesenchymal stem cell characterisation

Chondrogenic, adipogenic and osteogenic differentiation assays were performed and assessed as described previously [22].

### Metabolic activity

Cell suspensions from the needle gauge experiments were assayed for metabolic activity using the alamarBlue^®^ reagent. Cells were diluted to a concentration of 2.5 × 10^5^ cells/ml in D10, and 100 μl alamarBlue^®^ reagent was added and incubated at 37°C. At 4, 6, 8 and 24 hours, 100 μl aliquots of sample were then measured for fluorescence as detailed above. Readings obtained for needle injected cells were compared with values for the non-injected control cells.

### Apoptosis assay

The Annexin V–fluorescein isothiocyanate enzyme-linked immunosorbent assay (Cayman Chemical, Ann Arbor, MI, USA) was used to assess the effect of needle size on induction of apoptosis. The assay was validated in preliminary assays for cross-reactivity of the Annexin V antibody to the equine antigen using positive inducers of apoptosis. Cells were treated with staurosporine (500 and 100 nM) and incubated at 37°C for 3 hours or with sodium azide (20 nM) and incubated at 37°C for 2 hours. Cells were also heated to 45°C for 10 minutes to induce apoptosis. In addition, test cells were included where 1 ml cells in a 2 ml syringe were extruded through a 21G needle, and non-injected cells were suspended in D10 or in fresh allogeneic plasma. The assay was performed according to the manufacturer’s instructions*.* Briefly, staining solutions were prepared in Annexin V binding buffer (0.01 M HEPES, pH 7.4; 0.14 M NaCl; 2.5 mM CaCl_2_) to contain either Annexin V–fluorescein isothiocyanate antibody or propidium iodide, and were protected from direct light. Samples (100 μl containing 500,000 cells) were washed twice in 1 ml Annexin V binding buffer by centrifugation at 350 × *g* and cells were resuspended by gentle agitation in 250 μl of each staining solution. Samples were wrapped in aluminium foil to protect from direct light and incubated for 10 minutes at room temperature. Cells were washed as before and resuspended in 100 μl Annexin V binding buffer, and 10 μl aliquots were applied to a glass slide and mounted under a coverslip to visualise by fluorescence microscopy (using an Olympus BX61). The number of cells staining positive for propidium iodide (dead cells) using a rhodamine filter (excitation 540 nm and emission 570 nm) or Annexin V (cells in early apoptosis) using a fluorescein filter (excitation 485 nm and emission 535 nm) were counted. Means of cell counts from six fields of view per slide were determined at 20× magnification for both stains and the total cell number under a bright field. Test groups were analysed at 0, 2, 4 and 24 hours post injection.

**Figure 1:**
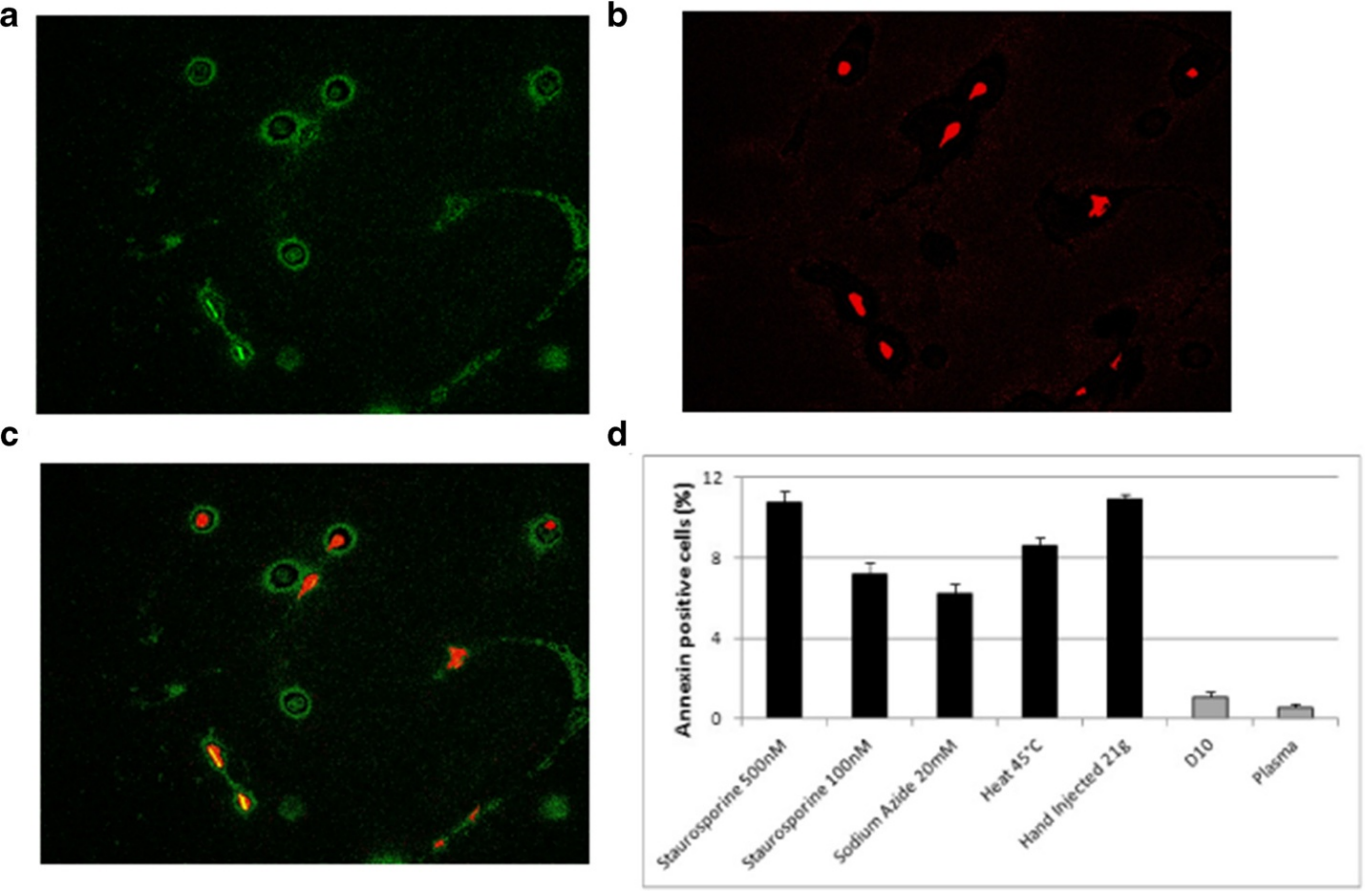
**Validation of apoptosis in equine mesenchymal stem cells.** Representative fluorescence images are shown for cells injected through a 21G needle, with **(a)** cell surface Annexin V (fluorescein isothiocyanate) and **(b)** nuclear propidium iodide staining of cells. **(c)** Merged Annexin V and propidium iodide images to distinguish coincidal and noncoincidal staining of cells. Cells in early apoptosis (green and green–red) and in late apoptosis (red) are present. Original magnification × 100, scale bar = 200 μm. **(d)** Quantification of apoptotic cells after treatment with known inducers of apoptosis. Cells injected through a 19G needle in D10 or in plasma are shown together with non-injected controls. D10, Dulbecco’s modified Eagle’s medium supplemented with 10% foetal bovine serum.

The validation experiments confirmed that the Annexin V antibody (anti-human antibody) cross-reacted with the equine antigen and revealed that the strongest inducers of apoptosis were 500 nM staurosporine and injection through the 19G needle. While all conditions induced apoptosis, there was no significant difference between any of the known apoptosis inducers. Comparison of the percentage of Annexin V immunofluorescent cells with the inducers of apoptosis with that of non-injected control cells in either D10 or fresh plasma revealed a significant increase in the proportion of early apoptotic cells with all apoptotic stimuli applied (*P* < 0.01; Figure 1).There was no significant difference in the mean total cell number between test groups, enabling confident comparison of the number of Annexin V-positive and propidium iodide-positive cells calculated as a percentage of total cells.

### Statistical analysis

Where descriptive analysis indicated that the data were not normally distributed, a log transformation was performed to normalise the data prior to analysis. Longitudinal data were analysed using a mixed-effects linear regression model in PASW (version 18; IBM, Portsmouth, UK), with Sidak correction. In order to interrogate the relationship between and within the multiple factors on cell viability or proliferation, statistical analysis was performed using analysis of variance within a mixed-effects model. *P* ≤0.05 was considered significant.

## Results

### Effect of resuspension media

**Figure 2:**
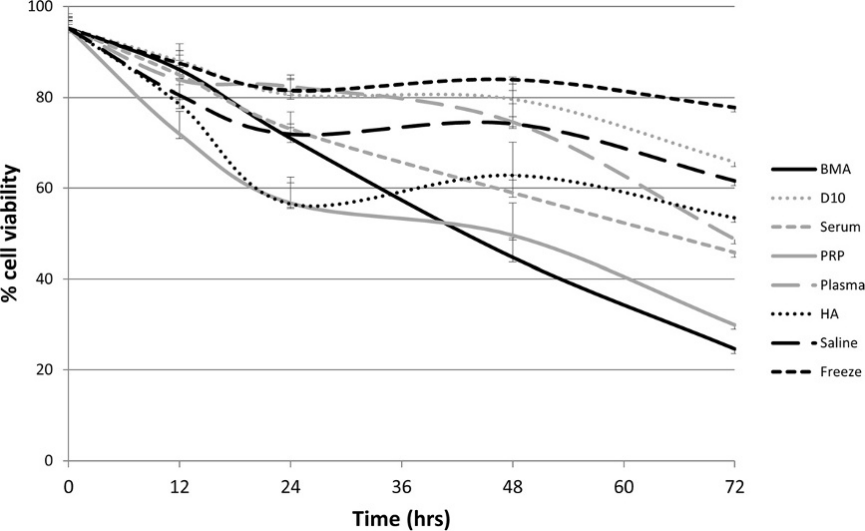
**Cell viability measured by trypan blue exclusion assay.** Viability of mesenchymal stem cells (*n* = 3) stored for up to 72 hours in a range of experimental suspension media are shown. BMA, bone marrow aspirate; D10, Dulbecco’s modified Eagle’s medium supplemented with 10% foetal bovine serum; HA, hyaluronic acid; PRP, platelet-rich plasma.

Cell viability was not significantly different between any of the storage media at 12 or 24 hours; however, viability decreased significantly over time (*P* < 0.001) and by 72 hours ranged from 25 to 78%, with the most rapid decline observed for cells suspended in the biological fluids, BMA, platelet-rich plasma and serum. Cell viability was greatest at all time points when cells were frozen in cryogenic medium. There was a significant reduction in viability for cells suspended in BMA in comparison with cells in cryogenic medium at 48 hours (*P* = 0.027) and 72 hours (*P* = 0.024) (Figure 2; Table 1).

**Table 1:** **Statistical comparison of cell viability over time in different suspension media**

Suspension media		***P*** value	Suspension media		***P*** value
BMA	D10	<0.001	D10	Serum	0.021
	Serum	n/s		PRP	<0.001
	PRP	n/s		Plasma	n/s
	Plasma	0.001		HA	n/s
	HA	0.01		Saline	n/s
	Saline	<0.001		Frozen	n/s
	Frozen	<0.001	Serum	PRP	n/s
PRP	Plasma	0.045		Plasma	n/s
	HA	n/s		HA	n/s
	Saline	0.007		Saline	n/s
	Frozen	<0.001		Frozen	0.001
Plasma	HA	n/s	HA	Saline	n/s
	Saline	n/s		Frozen	n/s
	Frozen	n/s	Saline	Frozen	n/s

BMA, bone marrow aspirate; D10, Dulbecco’s Modified Eagle Medium supplemented with 10% foetal bovine serum; HA, hyaluronic acid; n/s, no significant difference; PRP, platelet-rich plasma.

**Figure 3:**
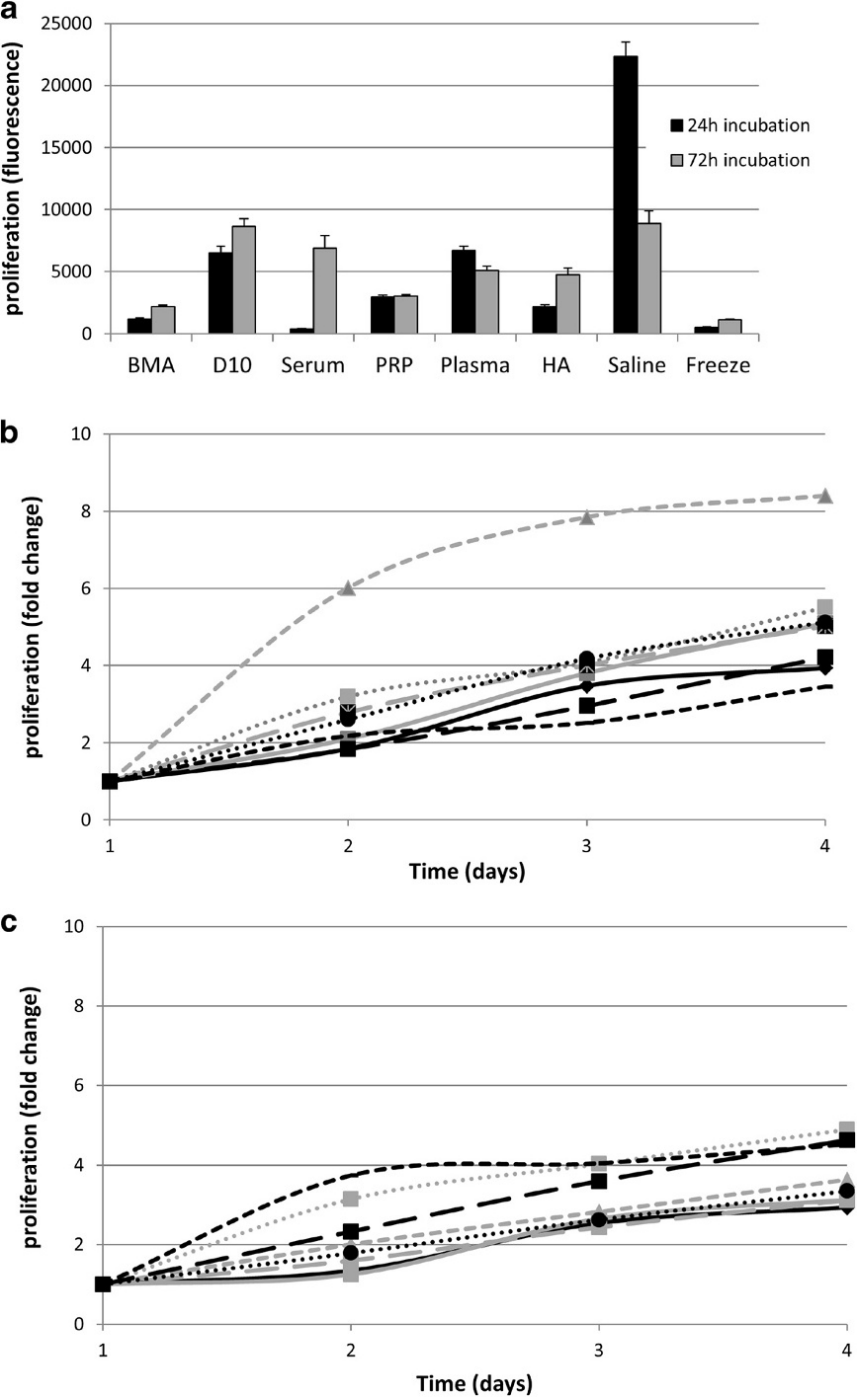
**Cell proliferation measured by alamarBlue** ^**®**^ **assay for mesenchymal stem cells (** ***n*** **= 3) following storage for 24 or 72 hours in suspension media. (a)** Initial proliferation rates. **(b)** Change in the proliferation rate with time following storage for 24 hours. **(c)** Change in the proliferation rate with time following storage for 72 hours. BMA, bone marrow aspirate; D10, Dulbecco’s modified Eagle’s medium supplemented with 10% foetal bovine serum; HA, hyaluronic acid; PRP, platelet-rich plasma. alamarBlue^®^ from AbD Serotec (Kidlington, UK).

Cells stored for 24 hours in saline initially proliferated significantly faster than those stored in any other media (*P* < 0.001 in all comparisons) but there were no significant differences in cell proliferation following 72 hours of storage. Interestingly, proliferation was enhanced at 72 hours compared with 24 hours for cells stored in BMA, D10, serum, hyaluronic acid and cryogenic medium, while other storage media showed a decrease (Figure 3a). Cells stored in all media showed similar proliferation rates when transferred to standard culture media (D10) except for those that had been stored in serum for 24 hours, in which there was a subsequently enhanced proliferation rate (Figure 3b,c).

### Effect of needle gauge on cell viability

**Figure 4:**
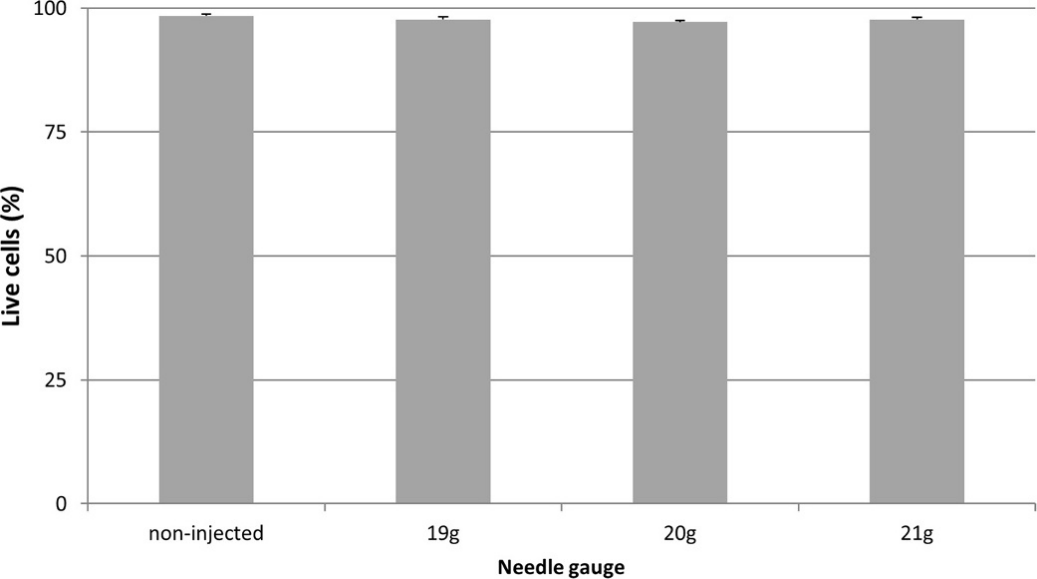
**Viability of cells after injection through different gauge needles.** Data are shown for viability measured immediately after injection of mesenchymal stem cells (*n* =3) in D10 through 19G, 20G or 21G needles. Non-injected control cells are also shown. There were no significant differences between any of the conditions. D10, Dulbecco’s modified Eagle’s medium supplemented with 10% foetal bovine serum.

**Figure 5:**
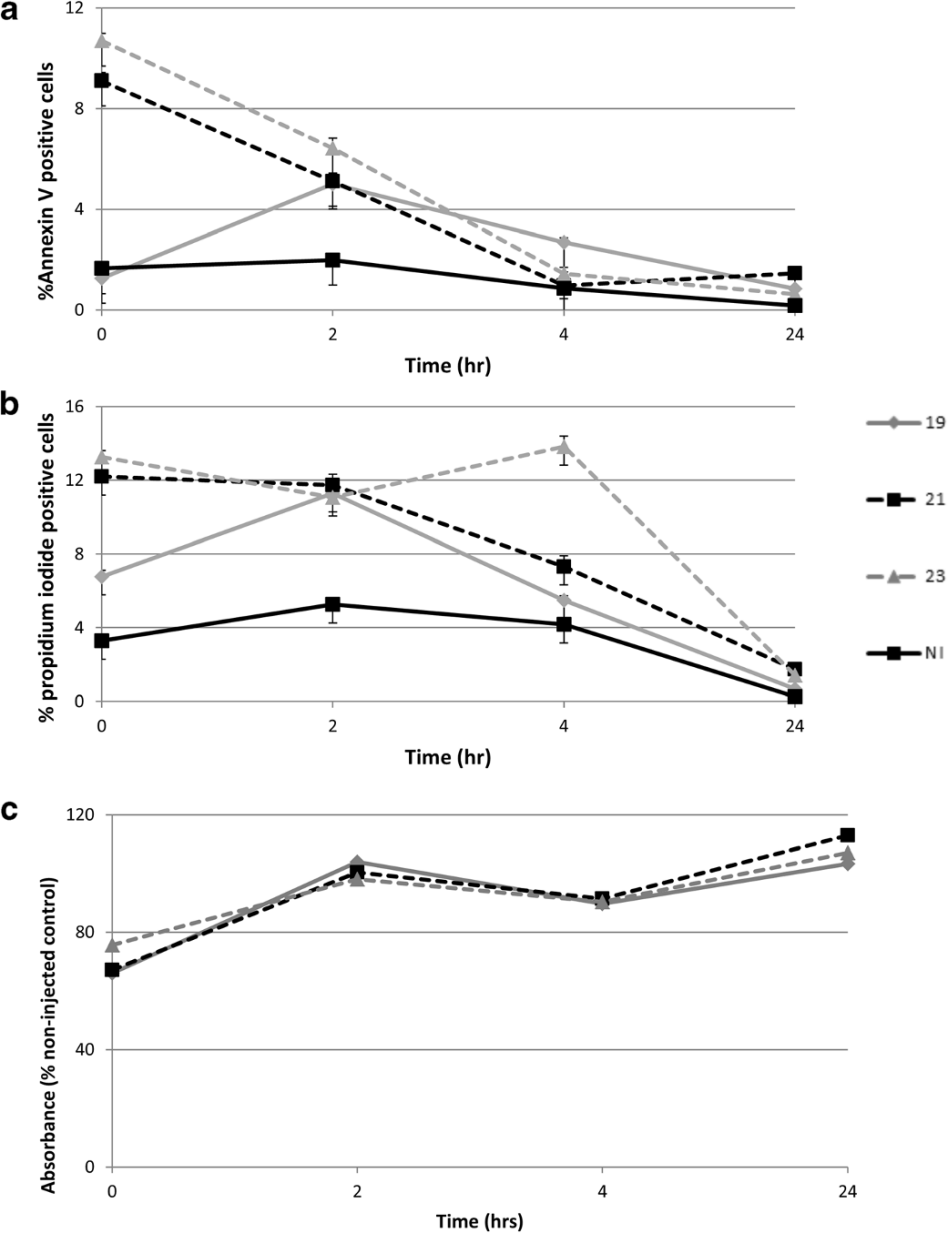
**Analysis of early and late apoptosis and cell metabolic activity after injection through different gauge needles.** Data are shown for viability measured immediately after injection of mesenchymal stem cells (*n* =3) in D10 through 19G, 20G or 21G needles and in non-injected controls (NI). **(a)** Annexin V-positive cells expressed as a percentage of the total cell population. **(b)** Propidium iodide-positive cells expressed as a percentage of the total cell population. **(c)** Metabolic activity of mesenchymal stem cells measured by alamarBlue^®^ (AbD Serotec, Kidlington, UK) assay.

Injection of cells did not cause an immediate loss in viability either for different needle gauges (Figure 4) or when comparing injected and non-injected groups (data not shown). There was, however, a significant effect of needle gauge on the proportion of cells staining positive for Annexin V (early apoptosis) or propidium iodide (late apoptosis) (*P* < 0.01; Figure 5a,b). There was a significant increase in apoptotic cells when injected through a 21G or 23G needle for both Annexin V (*P* < 0.01) and propidium iodide (*P* < 0.05) compared with non-injected cells, although there was no significant difference between non-injected cells and those injected through a 19G needle. Furthermore, there was a significant increase in cells staining positive with Annexin V following injection through the 23G (smallest diameter) needles compared with the 19G (largest diameter) needles (*P* = 0.023). Injection through the 21G needle (which is currently recommended for clinical use) resulted in approximately 9% of the cell population staining positive for Annexin V immediately post injection.There was minimal change in the percentage of Annexin V and propidium iodide-positive cells within the non-injected group over the 24-hour period. While Annexin V-positive cells decreased in the 21G and 23G groups over the 24-hour period, it was interesting to note that an increased number of positive cells was evident at 2 hours in the 19G group (Figure 5b), suggesting delayed induction of apoptosis.

There was a significant decrease in alamarBlue^®^ absorbance for all injected cells when compared with non-injected cells immediately post injection (*P* < 0.01), indicating an adverse effect on cell metabolism, but by 2 hours the injected cells had recovered to control levels (Figure 5c).

### Effect of needle gauge on cell differentiation

**Figure 6:**
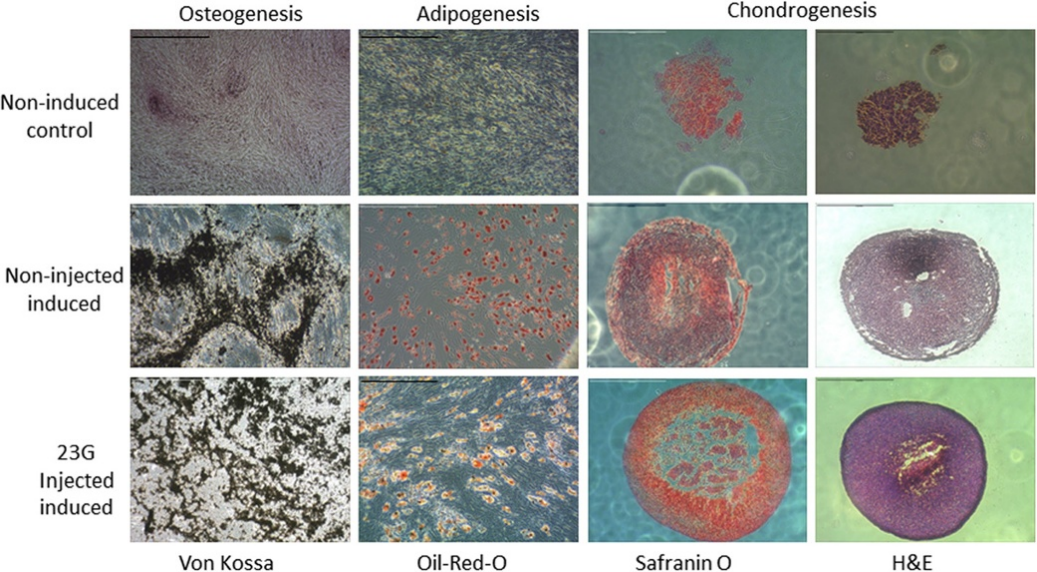
**Trilineage differentiation of mesenchymal stem cells.** Representative microscopic images of von Kossa and Oil-Red-O staining to confirm osteogenic and adipogenic differentiation of plastic adherent bone marrow-derived cell populations (scale bars = 100 μm). Chondrogenic differentiation was confirmed by the formation of dense cell pellets (haematoxylin and eosin (H&E) staining) that were positive for Safranin O (orange–red stain) (scale bars = 1 mm). Upper row: control, non-induced cultures; central row: control, induced (non-injected) cultures. Representative images for cell populations injected through a 23G needle and subsequently induced are shown. No difference was observed between these and populations injected through 20G or 21G needles.

MSCs all showed trilineage (osteogenic, adipogenic and lipogenic) differentiation and injection of cells did not result in any detectable alteration in differentiation ability either for different needle gauges or when comparing injected and non-injected groups (Figure 6).

## Discussion

This study has demonstrated significant adverse influences on MSCs associated with transport and implantation. It is therefore important to select a transport media and implantation technique that minimises these adverse effects so as to maximise viable cell delivery to the site of injury.

With respect to the selection of transport media, it would appear that any of the wide array of those tested would be satisfactory when implantation occurs within 24 hours, as is customary with the clinical supply of MSCs in the veterinary field. Once cells were suspended for longer than 24 hours, viability of cells stored in BMA was significantly less than if the cells were frozen in cryogenic medium. Although there is, as yet, no guidance on the storage of MSCs, it has been suggested that *in vitro* storage of haematopoietic stem or progenitor cells should not exceed 2 hours [23]. This suggestion is impractical both for current equine therapeutic use and when considering future, commercial applications of MSC therapy in other species, including humans, because of the necessity to transport the cells from a remote licensed facility to the clinic and our data suggest that up to 24 hours for transportation is possible without significant detrimental effects. For transportation periods suspected to be of greater duration than 24 hours, cells should ideally be frozen, to maximise cell viability. However, the cell suspension media in which MSCs were frozen is not an ideal carrier for intralesional injection, and resuspension in an alternative media would be necessary prior to use. This additional handling step in the clinic could be hazardous for cell survival and iatrogenic infection, and therefore a protocol for transportation periods longer than 24 hours requires further optimisation.

Although the reduction in cell viability was small, *in vivo* delivery of an increased number of dead cells potentially has two adverse consequences: first, a reduced efficacy; and second, the presence of dead cell debris, which may induce inflammation. While the first consequence can be compensated for by higher cell numbers, this compounds the problems of the second.

Human MSCs stored in saline at either 4°C or room temperature showed rapidly decreasing colony-forming unit frequency as storage times exceeded 1 hour, despite a population viability of over 90%, indicating that simple measurement of viability may not fully reflect either the self-renewal or proliferation capacity of MSCs [24]. Additionally, the differentiation potential (assessed by trilineage differentiation) gradually decreased over time, regardless of storage temperature, when compared with that of freshly harvested MSCs from culture [24]. This study therefore utilised more than one assessment of cellular health to evaluate effects other than immediate cell death. Other studies have reported that viability and osteogenic potential of human MSCs were not significantly altered by storage for 24 hours in phosphate-buffered saline [25] and that storage of up to 6 hours in an electrolyte solution (plasmalyte A) had no significant adverse effect on human MSCs, as measured by viability, differentiation capability and expression of cell surface markers [26]. These studies suggest that a loss in differentiation function is not an insurmountable, inevitable consequence of storage or shipment. In the current study, cells suspended in physiologic saline maintained surprisingly good viability and high rates of proliferation following 24 hours of storage, which is in line with previously reported, short-term viability in saline [24].

The reason for a more rapid rate of cell death in cells suspended in BMA, in comparison with saline or plasma, is not clear. The acellular fraction of bone marrow aspirate contains various growth factors and cytokines, and its composition may gradually alter during storage, with degradation of some beneficial growth factors and a gradual deterioration in quality, with a correspondingly negative influence on cell viability.

The current protocol for treating clinical tendon injuries involves a combination of MSCs and BMA [1, 9] although the relative contributions of each to tendon healing have not been clarified. Maintenance of the current protocol, which has been demonstrated to be both safe and efficacious [9] with increased emphasis on the importance of implantation within 24 hours of dispatch, should ensure sufficient cells remain viable.

This study utilised allogeneic BMA, serum and plasma, due to the practical and ethical difficulties in securing autologous fluid from each horse for which MSCs were available for experimental use. Future clinical use of allogeneic serum-containing transport media may necessitate heat inactivation of complement to further optimise cell viability. Pilot studies performed in our laboratory suggest that heat treatment of allogeneic plasma, bone marrow aspirate and platelet-rich plasma (56°C for 1 hour) results in a small, but measurable, improvement in cell viability (J. Dudhia, unpublished observations). The particular role of complement in cell death observed in this study is unknown and further work should be carried out to investigate this effect.

We have demonstrated that although the needle size did not have an effect on immediate cell viability, or on differentiation potential, there were significant effects on cellular processes. A cell damaged, but not killed, by the process of injection could enter one of three states: viable with a desired phenotype (that is, capable of differentiation and proliferation), surviving but dysfunctional, or apoptotic [18]. Dysfunctional cells could be further subdivided into those that sustain mechanical stress but remain able to restore their original capabilities, those with permanent phenotypic change, and those that later become apoptotic. The decrease in cell metabolic activity observed immediately post injection indicates that the injection process does alter cell health, although the subsequent increase in cell metabolic activity by 2 hours post injection suggests that the cells have recovered their original capabilities, as shown in other studies [18]. Although it is likely that the mechanism of action of MSCs *in vivo* involves processes more complex and wider reaching than simple *in situ* differentiation, we have demonstrated that this facility remains intact following injection.

The proportion of early apoptotic cells present immediately post injection decreased over the subsequent 24 hours. This correlates with the early stage in apoptosis at which externalisation of cell membrane phosphatidylserine residues occurs and maximum Annexin V binding would be expected. Indeed, the time from initiation of apoptosis to completion can occur in as little as 2 to 3 hours [14], which may explain the rapid decrease in the proportion of early apoptotic cells from 4 hours post injection. Although we did not measure caspase 3 levels or activation in the present study, analysis of this protein in future studies may enable the apoptotic pathways to be consolidated in better detail.

The peak in early apoptotic cells for the 19G group occurred at the 2-hour time point, whereas the peak for the 21G and 23G groups was immediately post injection. This observation may represent a difference in the intensity of mechanical sheer stress between the needle gauges, and supports previous observations that an increase in needle diameter decreases cell damage in human MSCs [19]. The smaller 21G and 23G needles may exert sufficient cell stress to induce apoptosis immediately, whereas the 19G needle may induce a lesser and more limited stress responsible for fewer cells undergoing delayed apoptosis. Indeed, variations in the fluid flow shear stress caused by needle walls have been shown to induce subtle phenotypic alterations and alter cell fate [27].

## Conclusions

We have demonstrated that, even following optimisation of these two necessary processes, a degree of cell death and altered cell function, albeit temporary, is to be expected. These results have implications for the efficacy of intralesional injection of MSCs for tendon repair. Every effort should be made to ensure that cells are reimplanted within 24 hours of resuspension in the laboratory and a needle with a bore larger than 21G should be used for injection.

## Abbreviations

BMA: bone marrow aspirate
MSC: mesenchymal stem cell.
