# Erratum to: Viability of equine mesenchymal stem cells during transport and implantation

**DOI:** 10.1186/s13287-016-0423-z

**Published:** 2016-11-09

**Authors:** Elaine R. Garvican, Sandra Cree, Lydia Bull, Roger K. W. Smith, Jayesh Dudhia

**Affiliations:** The Royal Veterinary College, Clinical Sciences and Services, Hawkshead Lane, North Mymms, Hatfield, AL9 7TA UK

## Erratum

Following the publication of our article [1] the following minor errors and omissions were brought to our attention.

## Introduction

Throughout the manuscript, reference should have been made to Bone Marrow Supernatant (BMS) and not Bone Marrow Aspirate (BMA).

## Materials and Methods

### Apoptosis assay

Assays using Annexin V and propidium iodide were performed on separate samples (replicates) and not as repeated measures, a point that was not made clear in the original article. Separate aliquots of cells for each time point were kept in culture, without media changes, until assayed.

### Cell proliferation assay and Metabolic activity

In alamarBlue® experiments relating to transport media, we used the interpretation of proliferation because cells were cultured over several days, allowing time for cell proliferation to take place. In contrast, when interpreting viability assays following needle extrusion, we employed a metabolic activity interpretation because the cells were cultured for a short period (24 h, Fig. 5) when cell proliferation would not be a prominent feature. Measurements in both instances were of fluorescence.

## Results

Figure 3: The Key was accidently omitted. The correct figure is provided here (Fig. 3).

**Fig. 3:**
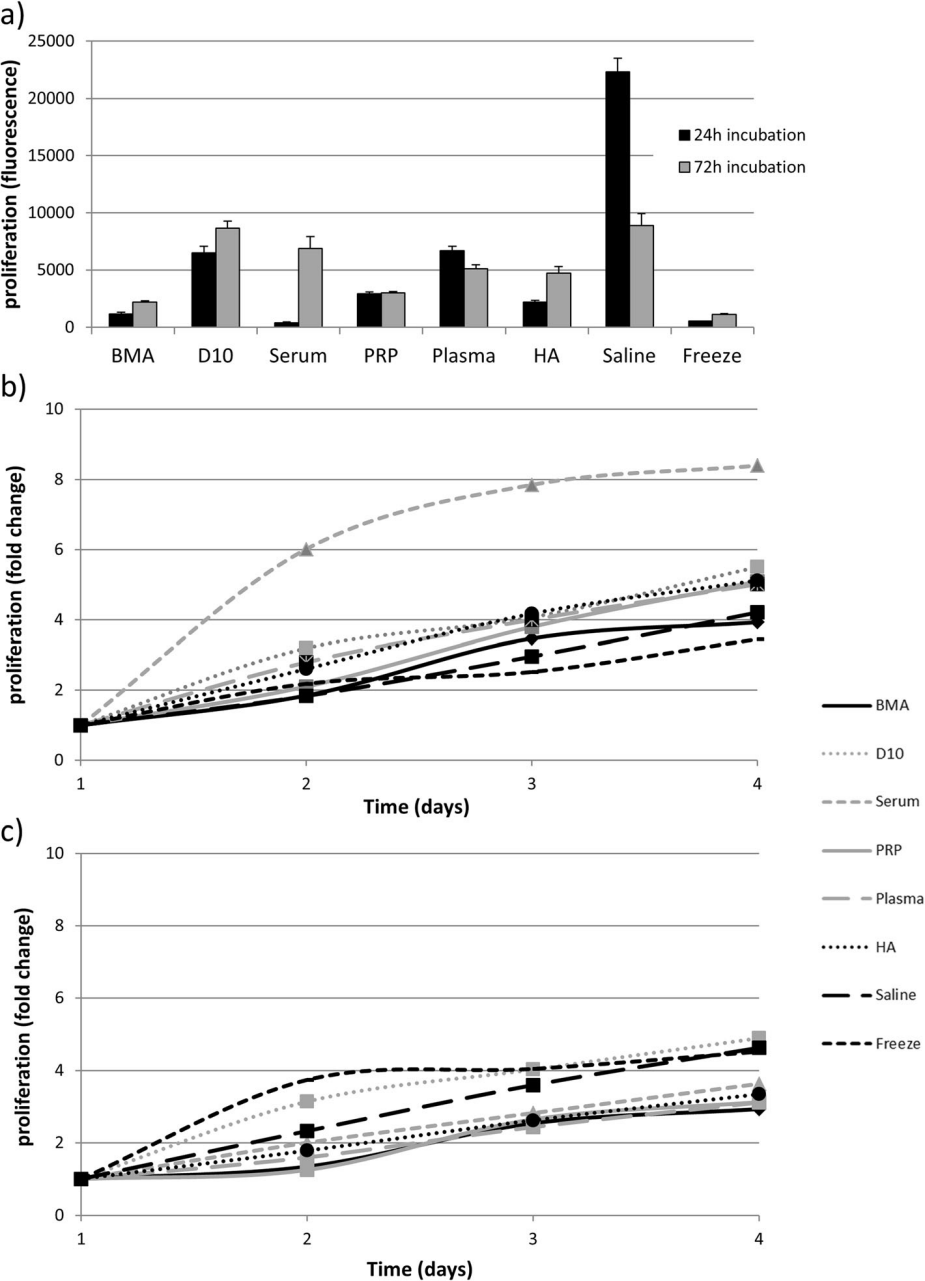
Cell proliferation measured by alamarBlue™ assay for mesenchymal stem cells (MSCs) (n = 3) following storage for 24 or 72 h in suspension media. **a** Initial proliferation rates; **b** Change in the proliferation rate with time following storage for 24 h; **c** Change in the proliferation rate with time following storage for 72 h. BMA, bone marrow aspirate; D10, Dulbecco’s Modified Eagle Medium supplemented with 10 % fetal bovine serum; PRP, platelet rich plasma; HA, hyaluronic acid. Error bars represent the standard error of the mean (± SEM)

Figure 5: The y-axis is incorrectly labelled as “Absorbance”. The correct axis label should read “% non-injected control” and is based on fluorescence readings. The correct figure is provided here (Fig. 5).

**Fig. 5:**
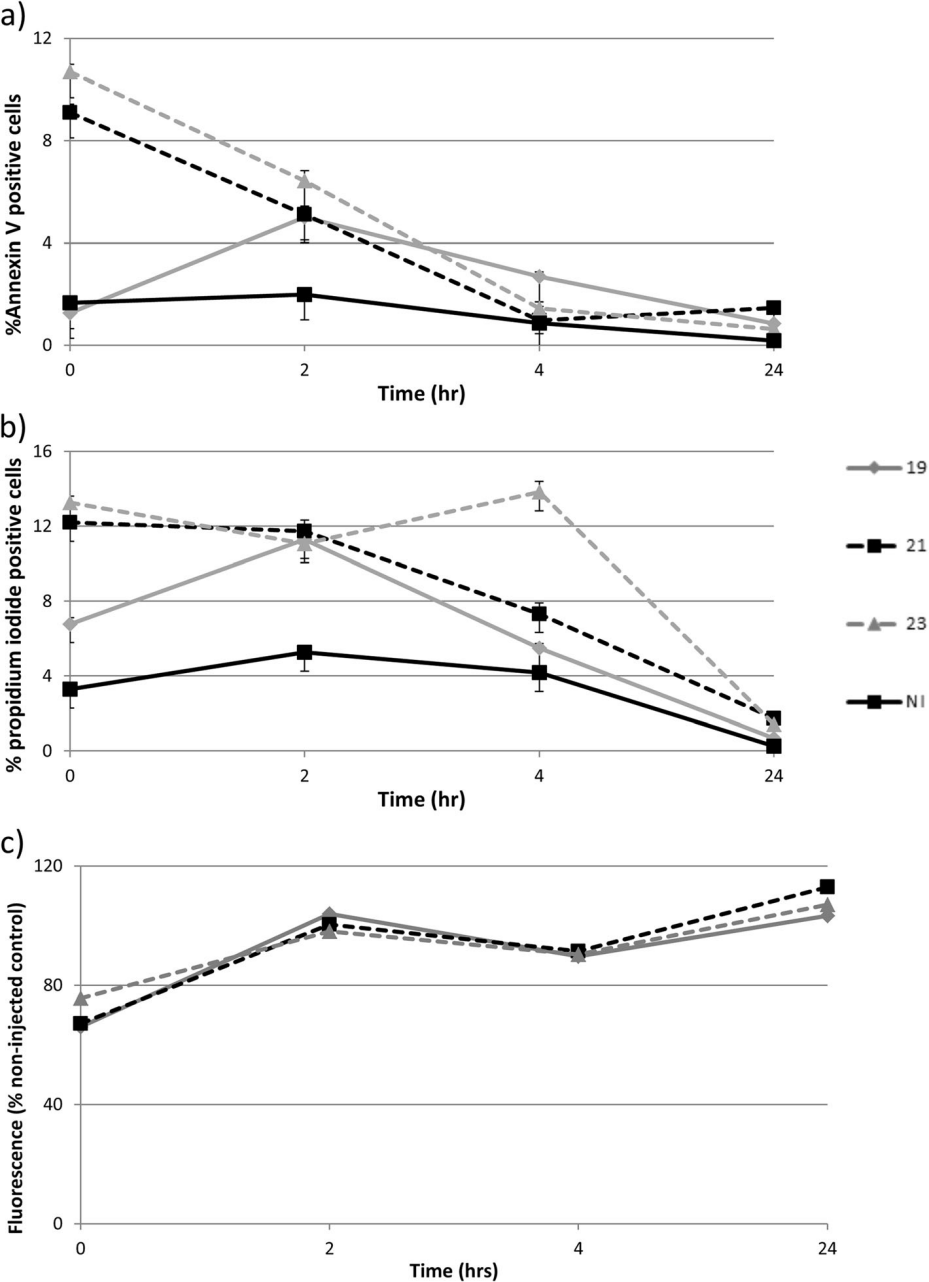
Analysis of early and late apoptosis and cell metabolic activity after injection through different gauge needles. Data is shown for viability measured immediately after injection of mesenchymal stem cells (n =3) in D10 through 19G, 20G or 21G needles and in non-injected controls (NI). **a**) Annexin V positive cells expressed as a percentage of the total cell population; **b** propidium iodide-positive cells expressed a percentage of the total cell population; **c** metabolic activity (fluorescence) of mesenchymal stem cells measured by alamarBlue™ assay. Error bars represent the standard error of the mean (± SEM)

Where appropriate, all figure legends in our article should state that error bars represent the standard error of the mean (± SEM).

These corrections do not alter the results or conclusion of the study that (i) the medium used for transportation of bone marrow mesenchymal stem cells had a significant impact on cell viability with increasing transport time and (ii) the use of a larger needle size (19G) for cell implantation reduced stress-induced loss of viability compared to smaller needle gauges (21G and 23G).
